# Patient Satisfaction With a Multisite, Multiregional Remote Patient Monitoring Program for Acute and Chronic Condition Management: Survey-Based Analysis

**DOI:** 10.2196/44528

**Published:** 2023-07-27

**Authors:** Tufia C Haddad, Karla C Maita, Jonathan W Inselman, Francisco R Avila, Ricardo A Torres-Guzman, Jordan D Coffey, Laura A Christopherson, Angela M Leuenberger, Sarah J Bell, Dominick F Pahl, John P Garcia, Lukas Manka, Antonio J Forte, Michael J Maniaci

**Affiliations:** 1 Center For Digital Health Mayo Clinic in Minnesota Rochester, MN United States; 2 Division of Hospital Internal Medicine Mayo Clinic in Florida Jacksonville, FL United States

**Keywords:** remote patient monitoring, patient satisfaction, postacute care, chronic conditions, acute conditions mobile phone

## Abstract

**Background:**

Remote patient monitoring (RPM) is an option for continuously managing the care of patients in the comfort of their homes or locations outside hospitals and clinics. Patient engagement with RPM programs is essential for achieving successful outcomes and high quality of care. When relying on technology to facilitate monitoring and shifting disease management to the home environment, it is important to understand the patients’ experiences to enable quality improvement.

**Objective:**

This study aimed to describe patients’ experiences and overall satisfaction with an RPM program for acute and chronic conditions in a multisite, multiregional health care system.

**Methods:**

Between January 1, 2021, and August 31, 2022, a patient experience survey was delivered via email to all patients enrolled in the RPM program. The survey encompassed 19 questions across 4 categories regarding comfort, equipment, communication, and overall experience, as well as 2 open-ended questions. Descriptive analysis of the survey response data was performed using frequency distribution and percentages.

**Results:**

Surveys were sent to 8535 patients. The survey response rate was 37.16% (3172/8535) and the completion rate was 95.23% (3172/3331). Survey results indicated that 88.97% (2783/3128) of participants agreed or strongly agreed that the program helped them feel comfortable managing their health from home. Furthermore, 93.58% (2873/3070) were satisfied with the RPM program and ready to graduate when meeting the program goals. In addition, patient confidence in this model of care was confirmed by 92.76% (2846/3068) of the participants who would recommend RPM to people with similar conditions. There were no differences in ease of technology use according to age. Those with high school or less education were more likely to agree that the equipment and educational materials helped them feel more informed about their care plans than those with higher education levels.

**Conclusions:**

This multisite, multiregional RPM program has become a reliable health care delivery model for the management of acute and chronic conditions outside hospitals and clinics. Program participants reported an excellent overall experience and a high level of satisfaction in managing their health from the comfort of their home environment.

## Introduction

The progressive increase in technology use in the health care environment has led to changes in the delivery of care to patients [1-3]. These shifts in the traditional in-person model of patient care toward telehealth have had a positive impact on patient recovery [4], hospital readmission, and length of stay [5,6], especially after the accelerated growth in the use of digital health technologies for internet-based, video consultations and remote patient monitoring (RPM) during the COVID-19 pandemic [7,8]. RPM programs proved to be a safe way to manage acute and chronic conditions during the pandemic, allowing health care systems to effectively navigate the hospital crisis and avoid overcrowded emergency rooms [9-11].

RPM has become a reliable option to manage care of patients in the comfort of their home or locations outside of hospitals and clinics [12]. Health care providers can continuously monitor patients’ physiological parameters and collect patient-reported symptom assessments using technology that can alert care teams to adverse health trends [13,14]. The timely intervention achieved with RPM models has been associated with a decrease in unnecessary hospitalizations and patient readmissions, proving an appropriate and cost-effective option for inpatient care [7,8,15,16]. Therefore, this approach has positively impacted the treatment of patients with chronic conditions, including congestive heart failure, hypertension or chronic obstructive pulmonary disease, as well as acute illness, as in the case of COVID-19 infection or postoperative care [17-20].

The Mayo Clinic initiated its RPM program in 2016 within its Midwest community-based health system. In 2018, the program was expanded to tertiary centers in the United States. Southeast (Jacksonville, FL), Midwest (Rochester, MN), and Southwest (Scottdale, AZ) regions. As of October 2022, approximately 22,000 patients have been served by this program. A fundamental part of this model of care is real-time data transmission, which permits care teams to gain insights into patient progress. Consequently, patients’ experience and level of satisfaction with the program play an essential role in their engagement and ability to accomplish successful clinical outcomes. Therefore, this study aimed to describe patients’ experiences and overall satisfaction with the multiregional, multisite Mayo Clinic RPM program.

## Methods

### Patient Population and Setting

The study was conducted between January 1, 2021, and August 31, 2022. Data used for the analysis were collected anonymously via a patient experience survey as a routine part of clinical practice from patients who participated in the RPM program at one of the 3 regional Mayo Clinic tertiary medical centers or one of the >70 rural, community-based Mayo Clinic Health System sites serving Western Wisconsin, Southern Minnesota, and Northern Iowa. Patient participation was voluntary, and any responder could withdraw from the survey at any time. The inclusion criteria for this study were as follows: (1) patients who had been accepted to the RPM program in the Mayo Clinic Health System or tertiary campuses in Arizona, Florida, and Minnesota; (2) patients who had a listed and functional email address; and (3) patients who completed the web-based survey. Patients were excluded if they did not have or list an email address to contact, if they refused to participate in the survey, or if the survey was returned unfilled. All surveys sent were completed after the patients were completely discharged from the RPM program. An email with a link to the survey was sent to each patient who was completely discharged from the RPM program.

### Background: The RPM Program

The Mayo Clinic RPM program offers a way for nurses, advanced practice providers, and physicians to deliver care to patients outside traditional hospital and clinic settings. It facilitates continuous communication between patients and providers through cellular connections, integrated vital sign monitors, videoconferencing, phone calls, and digitally delivered questionnaires to gather subjective patient-reported symptom assessments and objective physiological data. The provided technology allows for real-time collection and transmission of patient-generated health data, enabling a centralized team of RPM nurses to virtually monitor patient progress and detect health issues early through predefined parameters that trigger alerts. Nurses use clinical decision trees to manage patient care and escalate to the supervising provider to facilitate prompt diagnostic and treatment interventions.

The RPM program focuses on specific morbidities, with the goal of reducing hospital admission, readmission, and length of stay. RPM programs developed and implemented to date include those focused on chronic conditions such as coronary artery disease, congestive heart failure, chronic obstructive pulmonary disease, type 2 diabetes, uncontrolled hypertension, cirrhosis, as well as acute conditions such as acute COVID-19 infection, acute kidney injury, and febrile neutropenia [14,16,21,22]. In addition, the program supports patients following procedures such as thoracic surgery, coronary artery bypass graft, acute myocardial infarction or percutaneous intervention, chimeric antigen receptor T-cell therapy, and postintensive care unit. Patients within the Mayo Clinic system are eligible if they have a supported condition, are willing to use technology actively, are English-speaking, and are ≥18 years. Conversely, patients with uncontrolled mental health conditions, identified as end-of-life by the provider, with a limited ability to use RPM equipment or interact with staff, are not eligible for the program.

Once enrolled, patients receive a technology kit, which includes a cellular-enabled smart device (tablet or smartphone) paired with Bluetooth-enabled wearable or peripheral devices, such as a pulse oximeter, blood pressure monitor, thermometer, and weight scale. The collected data were transferred to a secure cloud-hosted platform and integrated into electronic health records. Patients were provided with instructions on how to use the equipment through an onboarding phone call with nonclinical support staff and quick paper reference guides included in the kit. They also received condition-specific educational information either with their kit or separately mailed to their home addresses. Nurses reviewed the educational materials with the patients as needed throughout the program. For patients needing additional assistance, technical and nonclinical support staff are available via toll-free phone numbers to provide support. Depending on the patient’s needs, individualized program goals were established and a series of tasks were assigned to be completed within specific time frames. Generally, this is a short-term program aimed at supporting self-management and lasts for 4-12 weeks, depending on the underlying condition.

### Patient Survey Creation

The RPM team worked with the Mayo Clinic Department of Patient Experience to develop the survey used in this study. It consisted of 19 questions categorized into 4 main areas: comfort, equipment, communication, and overall experience. In addition, there were 2 open-ended questions (Textbox 1). All the questions used a Likert-like scale with response options of (1) strongly agree, (2) agree, (3) neither agree nor disagree, (4) disagree, and (5) strongly disagree. The final section of the survey consisted of 2 open-ended questions asking participants to comment on what impressed and disappointed them about their RPM program experience.

**Survey questions delivered to patients enrolled in the remote patient monitoring program.**

**Comfort**

- The Remote Patient Monitoring Program helped me feel comfortable managing my health at home.

**Equipment**

- The team explained how to use the equipment.
- The medical equipment was easy to use.
- The equipment helped in my care at home.
- I felt comfortable interacting with the team by phone or tablet.

**Communication**

- The team explained things to me in a way that was easy to understand.
- The team listened to my concerns.
- The team kept me informed about my care plan.
- I was able to reach a member of the team right away for any questions or concerns.
- The team promptly responded to my needs.
- The team explained when I should seek medical attention.
- The educational materials provided by the team were useful (ie, information packets, booklets, pamphlets, etc.)

**Overall experience**

- I felt ready to leave the Remote Patient Monitoring program.
- The team treated me with courtesy and respect.
- The staff worked well together to care for me.
- I would recommend the Remote Patient Monitoring Program to others with a similar health condition(s).
- Overall, I am satisfied with the Remote Patient Monitoring Program.

**Personal comments**

- Please tell us what impressed you about the Remote Patient Monitoring Program.
- Please tell us what disappointed you about the Remote Patient Monitoring Program.

To ensure the effectiveness of the survey, the initial draft was reviewed by a clinical team. They provided feedback on the language used in the survey, the relevance of each survey question, and whether the patients would be able to provide appropriate answers. The survey was edited based on this feedback and the final draft was emailed to the patients.

### Data Collection and Statistical Analysis

This study was a retrospective review of patient experience surveys conducted over a fixed period. As such, no power analysis or sample size calculations were performed, and all returned surveys within the designated time frame were included in the analysis. In addition to the survey responses, the study team also gathered patient demographic characteristics, including age, sex, race, and ethnicity, as well as the specific conditions for which the patient required RPM. The patients accessed the secured survey through an email link, and all study data were collected and managed using electronic data capture tools hosted at the Mayo Clinic.

The analysis of the Likert questions involved standard descriptive statistics, including frequency distribution and percentages. In addition, the mean score for each question was calculated, and CIs were determined using the mean CI formula based on the sample SD. The Shapiro-Wilk test was used to assess whether the observed Likert scores were normally distributed. No qualitative or data saturation analysis was performed on the freeform answers; common answers were grouped and reported in a descriptive fashion. Survey responses were compared between the levels of demographic variables for patient sex, age group, marital status, and education level. To ensure an adequate sample size at each level, survey responses were combined into 2 categories: agree positive responses (strongly agree and agree) were combined and negative or indifferent responses were combined (neither agree nor disagree, disagree, and strongly disagree). Differences were assessed using the chi-square test, and a *P* value of ≤.05 was considered statistically significant. The analysis was conducted using SAS (version 9.04; SAS Institute Inc).

### Ethics Approval

This study was approved by the Mayo Clinic Institutional Review Board, under protocol number 18-009605, with a waiver of informed consent.

### Data Storage and Security

Patient characteristic data were stored and protected on the Mayo Clinic electronic health records and internal servers. Surveys were distributed securely using a vetted survey tool, enabling researchers to automate survey delivery to all patients who completed the program. The survey tool automatically generates a unique survey link for each patient, enabling the comparison of survey responses to other patient data such as demographics and program type.

## Results

### Patients and Survey Response and Completion Rates

Of the 12,172 patients enrolled in the RPM at the Mayo Clinic between January 1, 2021, and August 31, 2022, a total of 8535 (70.12%) had a listed and operational email address to receive a survey. A total of 3331 patients started the survey, and 3172 completed it, representing a response rate of 37.16% and a completion rate of 95.23%, respectively. The analysis cohort comprised patients who completed the survey. The cohort included 49.09% (1557/3172) male and 50.91% (1615/3172) female. On the basis of race and ethnicity, the patient cohort was 95.96% (3044/3172) White, 2.43% (77/3172) Hispanic or Latino, 1.51% (48/3172) Black or African American, and 1.2% (38/3172) Asian. The average age of the respondents was 66.6 with an SD of 12.7 (Table 1).

The RPM programs included those for eligible conditions (Table 2). Most RPM program participants were patients with a diagnosis of COVID-19 at risk for severe disease, followed by patients with chronic conditions, such as uncontrolled hypertension and congestive heart failure. The lower volume programs were more recently implemented specialty programs involving the postintensive care unit, febrile neutropenia for cancer, and acute kidney injury.

The survey completion rates varied between sections of the survey, with structured questions associated with the highest rate (95.59%-98.61%) and open-ended questions representing the lowest completion rates (72.24% and 61.54% when responding to what was impressed and disappointed about the RPM program, respectively). The overall results of the structured survey are presented in Table 3**.**

**Table 1:** Patient demographic characteristics.

			Arizona (n=479)		Florida (n=220)		Minnesota (n=2366)		Wisconsin (n=107)		Total (N=3172)
**Sex, n (%)**											
	Male	296 (61.8)		119 (54.1)		1140 (48.18)		60 (56.1)		1557 (49.09)	
	Female	183 (38.2)		101 (45.9)		1226 (51.82)		47 (43.9)		1615 (50.91)	
Age (years), mean (SD)			65.6 (12.9)		65.9 (12.6)		66.6 (12.6)		72.6 (11.9)		66.6 (12.7)
**Race, n (%)**											
	American Indian or Alaskan native	6 (1.3)		1 (0.5)		8 (0.34)		1 (0.9)		16 (0.5)	
	Asian	10 (2.1)		8 (3.6)		20 (0.85)		0 (0)		38 (1.2)	
	Black or African American	10 (2.1)		14 (6.4)		23 (0.97)		1 (0.9)		48 (1.51)	
	Choose not to disclose	1 (0.2)		3 (1.4)		7 (0.3)		0 (0)		11 (0.35)	
	Native Hawaiian or Pacific Islander	1 (0.2)		0 (0)		1 (0.04)		0 (0)		2 (0.06)	
	Other	1 (0.2)		2 (0.9)		7 (0.3)		0 (0)		10 (0.36)	
	Unknown	1 (0.2)		0 (0)		2 (0.08)		0 (0)		3 (0.09)	
	White	449 (93.7)		192 (87.3)		2298 (97.13)		105 (98.1)		3044 (95.96)	
**Ethnicity, n (%)**											
	Hispanic or Latino	46 (9.6)		7 (3.2)		23 (0.97)		1 (0.9)		77 (2.43)	
	Choose not to disclose	3 (0.6)		3 (1.4)		31 (1.31)		0 (0)		37 (1.17)	
	Not Hispanic or Latino	427 (89.1)		210 (95.5)		2306 (97.46)		106 (99.1)		3049 (96.12)	
	Unknown	3 (0.6)		0 (0)		6 (0.25)		0 (0)		9 (0.28)	

**Table 2:** RPM^a^ program participation by eligible condition among survey respondents.

RPM programs	Participation (N=3172), n (%)
Acute kidney injury	7 (0.22)
Coronary artery disease	24 (0.76)
CAR-T^b^	25 (0.79)
Cirrhosis	8 (0.25)
General complex care	13 (0.41)
COPD^c^	21 (0.7)
COVID-19	2795 (88.11)
Congestive heart failure	81 (2.55)
Hypertension	87 (2.74)
Febrile neutropenia	3 (0.09)
Other	9 (0.28)
Post-ICU^d^	1 (0.03)
Postsurgical	98 (3.09)

^a^RPM: remote patient monitoring.

^b^CAR-T: chimeric androgen receptor T-cell therapy.

^c^COPD: chronic obstructive pulmonary disease.

^d^Post-ICU: postintensive care unit.

**Table 3:** Remote patient monitoring program patient experience survey questions and responses.

Questions (number of respondents)			Answers (number of respondents), n (%)											Mean (95% CI)^a^			*P* value^b^
			Strongly agree		Agree		Neither agree or disagree		Disagree		Strongly disagree						
**Comfort**																	
	The Remote Patient Monitoring Program helped me feel comfortable managing my health at home (n=3128, 98.61%)	1677 (53.61)		1106 (35.36)		188 (6.01)		33 (1.05)		124 (3.96)		4.3462 (4.3139-4.3785)			<.001		
**Equipment**																	
	The team explained how to use the equipment (n=3099, 97.7%)	1697 (54.76)		1047 (33.79)		214 (6.91)		69 (2.23)		72 (2.32)		4.3653 (4.3341-4.3965)			<.001		
	The medical equipment was easy to use (n=3079, 97.07%)	1913 (62.13)		945 (30.69)		80 (2.6)		65 (2.11)		76 (2.47)		4.4778 (4.4476-4.508)			<.001		
	The equipment helped in my care at home (n=3076, 96.97%)	1638 (53.25)		1039 (33.78)		285 (9.27)		53 (1.72)		61 (1.98)		4.3511 (4.3208-4.3814)			<.001		
	I felt comfortable interacting with the team by phone or tablet (n=3081, 97.13%)	1962 (63.68)		935 (30.35)		106 (3.44)		22 (0.71)		56 (1.82)		4.5345 (4.5078-4.5612)			<.001		
**Communication**																	
	The team explained things to me in a way that was easy to understand (n=3079, 97.07%)	1824 (59.24)		1038 (33.71)		140 (4.55)		22 (0.71)		55 (1.79)		4.4779 (4.4506-4.5052)			<.001		
	The team listened to my concerns (n=3065, 96.63%)	1816 (59.25)		951 (31.03)		230 (7.5)		20 (0.65)		48 (1.57)		4.4630 (4.4354-4.4906)			<.001		
	The team kept me informed about my care plan (n=3063, 96.56%)	1602 (52.3)		1035 (33.79)		303 (9.89)		74 (2.42)		49 (1.6)		4.3289 (4.2982-4.3596)			<.001		
	I was able to reach a member of the team right away for any questions or concerns (n=3039, 95.81%)	1523 (50.12)		947 (31.16)		477 (15.7)		48 (1.58)		44 (1.45)		4.2662 (4.2347-4.2977)			<.001		
	The team promptly responded to my needs (n=3038, 95.78%)	1652 (54.38)		960 (31.6)		345 (11.36)		33 (1.09)		48 (1.58)		4.3581 (4.3281-4.3881)			<.001		
	The team explained when I should seek medical attention (n=3032, 95.59%)	1597 (52.67)		937 (30.9)		412 (13.59)		40 (1.32)		46 (1.52)		4.3218 (4.2911-4.3525)			<.001		
	The educational materials provided by the team were useful (n=3054, 96.28%)	1391 (45.55)		1192 (39.03)		397 (13)		30 (0.98)		49 (1.44)		4.2635 (4.2342-4.2928)			<.001		
**Overall experience**																	
	I felt ready to leave the Remote Patient Monitoring program (n=3077, 97.01%)	1898 (61.68)		997 (32.4)		88 (2.86)		45 (1.46)		49 (1.59)		4.511 (4.4839-4.5381)			<.001		
	The team treated me with courtesy and respect (n=3070, 96.78%)	2246 (73.16)		736 (23.97)		34 (1.11)		10 (0.33)		44 (1.43)		4.8919 (4.6501-4.6965)			<.001		
	The staff worked well together to care for me (n=3063, 96.56%)	1974 (64.45)		834 (27.23)		182 (5.94)		21 (0.69)		52 (1.7)		4.5206 (4.4928-4.5484)			<.001		
	I would recommend the Remote Patient Monitoring Program to others with a similar health condition(s); n=3068, 96.72%)	2049 (66.7)		797 (25.98)		129 (4.2)		34 (1.11)		59 (1.92)		4.5469 (4.5189-4.5749)			<.001		
	Overall, I am satisfied with the Remote Patient Monitoring Program (n=3070, 96.78%)	2026 (65.99)		847 (27.59)		107 (3.49)		34 (1.11)		56 (1.82)		4.5492 (4.5218-4.5766)			<.001		

^a^Calculated by CI calculator.

^b^Calculated by the Shapiro-Wilk test.

### Survey Results

The survey included 19 questions divided into 4 categories. The first category was related to how comfortable respondents felt while managing their health at home using the RPM program. Overall, patients responded positively, with 88.97% (2783/3128) patients answering, “strongly agree” or “agree.”

The second category evaluated equipment performance and its utility for health care team interactions. The average response rate for this category was 97.23% (3084/3172). In total, 88.54% (2744/3099) patients “strongly agree” or “agree” when asked about receiving instructions from the health team about how to use the equipment. Of the 3079 patients, 92.82% (2858/3079) “strongly agree” or “agree” that the medical equipment was easy to use, with 87.03% (2677/3079) respondents also positively agreeing that the equipment helped in their care at home. Patients reported feeling comfortable interacting with the RPM team using a phone or tablet with a rate of agreement represented by 94.03% (2897/3081) of patients who “strongly agree” or “agree” to this question.

The effectiveness of communication as part of the RPM program was assessed in the third category, with an average response rate of 96.25% (3053/3172). The 7 questions used in this category demonstrated that most patients “strongly agree” or “agree.” Specifically, 59.24% (1824/3079) patients strongly agreed and 33.71% (1038/3079) agreed that the RPM team explained the program in a way that was easy to understand; 59.25% (1816/3065) of respondents strongly agreed and 31.03% (951/3065) agreed that the RPM team listened to their concerns; and 54.38% (1652/3038) strongly agreed and 31.6% (960/3038) agreed that the RPM team promptly responded to their needs.

In addition, 52.3% (1602/3063) patients strongly agreed, and 33.71% (1035/3063) agreed when asked if the team kept them informed about their health care plan. Patients also agreed or strongly agreed that the team explained when they should seek medical attention in 30.9% (937/3032) and 52.67% (1597/3032) of the cases, respectively. However, a moderate percentage of RPM patients were neutral (477/3039, 15.7%) or in disagreement (92/3039, 3.03%) when evaluating their ability to reach a member of the team immediately for questions or concerns. A similar percentage of patients disagreed or were neutral when asked about the usefulness of the educational material provided by the RPM team.

The fourth category comprised overall experience questions related to staff, sense of achievement with the program, and satisfaction. Of the 3070 patients, the majority strongly agreed (2246/3070, 73.16%) or agreed (736/3070, 23.97%) that the team treated them with courtesy and respect. Only 1.76% (54/3070) of the participants strongly disagreed or disagreed with this question. When asked about teamwork performance, 91.67% (2808/3063) of patients strongly agreed or agreed that the staff worked well together, taking care of them. Finally, the participants demonstrated a high degree of satisfaction with 93.58 % (2873/3070) of patients choosing “strongly agree” or “agree” options. When asked if they would recommend the RPM program to others with similar conditions, 92.76% (2846/3068) of patients strongly agreed or agreed, and only 3.03% (93/3068) strongly disagreed or disagreed.

### Association of Demographic Variables With Program Satisfaction

Patient satisfaction with the RPM was evaluated using the variables of sex, age, education level, and marital status. Some significant differences were observed between groups. As presented in Table 4, a lower percentage of female than male patients indicated agreement that the team explained how to use the equipment (1324/1516, 87.34% vs 1420/1583, 89.7%; *P*=.04) or that they were able to reach a team member immediately for questions or concerns (1185/1491, 79.48% vs 1285/1548, 83.01%; *P*=.01).

Considering patients’ age, significant differences were observed in the 2 aspects of the program (Table 5). First, the youngest age group (aged 18-34 years) had significantly lower agreement than other age groups that the equipment helped in their care at home. Second, the oldest age group (age ≥75 years) had the lowest agreement that the team explained when they should seek medical attention in comparison with the youngest age group (627/794, 79% vs 53/59, 90%; *P*<.001).

Marital status was significantly associated with overall experience, equipment use, and team communication (Table 6). Single patients expressed lower agreement that the equipment helped in their care at home (*P*=.03), the team listened to their concerns (*P*=.04), and overall satisfaction with the RPM program (*P*=.03). Notably, there were significantly fewer married survey respondents who were female than male (1094/1615, 67.7% vs 1307/1557, 83.94%), and the youngest age group was significantly less likely to be married (*P*<.001). There were no differences by marital status in comfort in managing their health at home, finding the equipment easy to use, and having positive interactions with the team.

Although program participants generally had positive experiences regardless of their level of education, important differences were identified (Table 7). Those with lower levels of education (high school or less) had higher agreement that the team kept them informed about their care plan (*P*=.02) and that the educational materials provided were useful (*P*=.01) when compared with those with higher levels of education. In addition, there was a higher level of agreement that the equipment helped in home care (*P*=.02) among those with a higher level of education.

**Table 4:** Remote Patient Monitoring program patient experience survey questions and responses according to patient sex.

Questions (number of respondents)				Female (n=1557), n (%)		Male (n=1615), n (%)		*P* value	
**Comfort**									
	**The Remote Patient Monitoring Program helped me feel comfortable managing my health at home (n=3128, 98.61%)**								.90^a^
		Total	1533 (100)		1595 (100)				
		Agree	1365 (89.04)		1418 (88.90)				
		Disagree	168 (10.96)		177 (11.10)				
**Equipment**									
	**The team explained how to use the equipment (n=3099, 97.7%)**								.04^a^
		Total	1516 (100)		1583 (100)				
		Agree	1324 (87.34)		1420 (89.70)				
		Disagree	192 (12.66)		163 (10.30)				
	**The medical equipment was easy to use (n=3079, 97.07%)**								.47^a^
		Total	1502 (100)		1577 (100)				
		Agree	1389 (92.48)		1469 (93.15)				
		Disagree	113 (7.52)		108 (6.85)				
	**The equipment helped in my care at home (n=3076, 96.97%)**								.48^a^
		Total	1508 (100)		1568 (100)				
		Agree	1319 (87.47)		1358 (86.61)				
		Disagree	189 (12.53)		210 (13.39)				
	**I felt comfortable interacting with the team by phone or tablet (n=3081, 97.13%)**								.23^a^
		Total	1510 (100)		1571 (100)				
		Agree	1412 (93.51)		1485 (94.53)				
		Disagree	98 (6.49)		86 (5.47)				
**Communication**									
	**The team explained things to me in a way that was easy to understand (n=3079, 97.07%)**								.16^a^
		Total	1505 (100)		1574 (100)				
		Agree	1389 (92.29)		1473 (93.58)				
		Disagree	116 (7.71)		101 (6.42)				
	**The team listened to my concerns (n=3065, 96.63%)**								.59^a^
		Total	1497 (100)		1568 (100)				
		Agree	1347 (89.98)		1420 (90.56)				
		Disagree	150 (10.02)		148 (9.44)				
	**The team kept me informed about my care plan (n=3063, 96.56%)**								.11^a^
		Total	1499 (100)		1564 (100)				
		Agree	1275 (85.1)		1362 (87.1)				
		Disagree	224 (14.9)		202 (12.9)				
	**I was able to reach a member of the team right away for any questions or concerns (n=3039, 95.81%)**								.01^a^
		Total	1491 (100)		1548 (100)				
		Agree	1185 (79.48)		1285 (83.01)				
		Disagree	306 (20.52)		263 (16.99)				
	**The team promptly responded to my needs (n=3038, 95.78%)**								.25^a^
		Total	1490 (100)		1548 (100)				
		Agree	1270 (85.23)		1342 (86.69)				
		Disagree	220 (14.77)		206 (13.31)				
	**The team explained when I should seek medical attention (n=3032, 95.59%)**								.08^a^
		Total	1479 (100)		1553 (100)				
		Agree	1218 (82.35)		1316 (84.74)				
		Disagree	261 (17.65)		237 (15.26)				
	**The educational materials provided by the team were useful (ie, information packets, booklets, pamphlets, etc.) (n=3054, 96.28%)**								.71^a^
		Total	1500 (100)		1554 (100)				
		Agree	1265 (84.33)		1318 (84.81)				
		Disagree	235 (15.67)		236 (15.19)				
**Overall experience**									
	**I felt ready to leave the Remote Patient Monitoring program (n=3077, 97.01%)**								.02^a^
		Total	1508 (100)		1569 (100)				
		Agree	1404 (93.10)		1491 (95.03)				
		Disagree	104 (6.90)		78 (4.97)				
	**The team treated me with courtesy and respect (n=3070, 96.56%)**								.20^a^
		Total	1503 (100)		1567 (100)				
		Agree	1454 (96.74)		1528 (97.51)				
		Disagree	49 (3.26)		39 (2.49)				
	**The staff worked well together to care for me (n=3063, 96.56%)**								.049^a^
		Total	1501 (100)		1562 (100)				
		Agree	1361 (90.67)		1447 (92.64)				
		Disagree	140 (9.33)		115 (7.36)				
	**I would recommend the Remote Patient Monitoring Program to others with a similar health condition(s), (n=3068, 96.72%)**								.69^a^
		Total	1504 (100)		1564 (100)				
		Agree	1392 (92.55)		1454 (92.97)				
		Disagree	112 (7.45)		110 (7.03)				
	**Overall, I am satisfied with the Remote Patient Monitoring Program (n=3070, 96.78%)**								.70^a^
		Total	1505 (100)		1565 (100)				
		Agree	1411 (93.75)		1462 (93.42)				
		Disagree	94 (6.25)		103 (6.58)				

^a^Chi-square *P* value.

**Table 5:** Remote patient monitoring program patient experience survey questions and responses by patient age group.

Questions (number of respondents)				Age group (years), n (%)											*P* value
				18-34 (n=59)		35-49 (n=264)		50-64 (n=840)		65-74 (n=1151)		≥75 (n=858)			
**Comfort**															
	**The Remote Patient Monitoring Program helped me feel comfortable managing my health at home (n=3128, 98.61%)**														.48^a^
		Total	59 (100)		262 (100)		830 (100)		1137 (100)		840 (100)				
		Agree	56 (94.9)		237 (90.5)		731 (88.1)		1014 (89.2)		745 (88.7)				
		Disagree	3 (5.1)		25 (9.5)		99 (11.9)		123 (10.8)		95 (11.3)				
**Equipment**															
	**The team explained how to use the equipment (n=3099, 97.7%)**														.59^a^
		Total	59 (100)		260 (100)		823 (100)		1128 (100)		829 (100)				
		Agree	55 (93.2)		228 (87.7)		723 (87.8)		1008 (89.4)		730 (88.1)				
		Disagree	4 (6.8)		32 (12.3)		100 (12.2)		120 (10.6)		99 (11.9)				
	**The medical equipment was easy to use (n=3079, 97.07%)**														.08^a^
		Total	59 (100)		261 (100)		814 (100)		1122 (100)		823 (100)				
		Agree	56 (94.9)		252 (96.6)		760 (93.4)		1036 (92.3)		754 (91.6)				
		Disagree	3 (5.1)		9 (3.4)		54 (6.6)		86 (7.7)		69 (8.4)				
	**The equipment helped in my care at home (n=3076, 96.97%)**														.004^a^
		Total	59 (100)		261 (100)		819 (100)		1118 (100)		819 (100)				
		Agree	44 (74.6)		237 (90.8)		727 (88.8)		968 (86.6)		701 (85.6)				
		Disagree	15 (25.4)		24 (9.2)		92 (11.2)		150 (13.4)		118 (14.4)				
	**I felt comfortable interacting with the team by phone or tablet (n=3081, 97.13%)**														.63^a^
		Total	59 (100)		261 (100)		820 (100)		1120 (100)		821 (100)				
		Agree	54 (91.5)		248 (95.0)		772 (94.1)		1058 (94.5)		765 (93.2)				
		Disagree	5 (8.5)		13 (5)		48 (5.9)		62 (5.5)		56 (6.8)				
**Communication**															
	**The team explained things to me in a way that was easy to understand (n=3079, 97.07%)**														.79^a^
		Total	59 (100)		260 (100)		822 (100)		1121 (100)		817 (100)				
		Agree	55 (93.2)		245 (94.2)		761 (92.6)		1047 (93.4)		754 (92.3)				
		Disagree	4 (6.8)		15 (5.8)		61 (7.4)		74 (6.6)		63 (7.7)				
	**The team listened to my concerns (n=3065, 96.63%)**														.68^a^
		Total	59 (100)		259 (100)		816 (100)		1121 (100)		810 (100)				
		Agree	53 (89.8)		227 (87.6)		738 (90.4)		1015 (90.5)		734 (90.6)				
		Disagree	6 (10.2)		32 (12.4)		78 (9.6)		106 (9.5)		76 (9.4)				
	**The team kept me informed about my care plan (n=3063, 96.56%)**														.25^a^
		Total	59 (100)		261 (100)		819 (100)		1115 (100)		809 (100)				
		Agree	52 (88.1)		226 (86.6)		719 (87.8)		961 (86.2)		679 (83.9)				
		Disagree	7 (11.9)		35 (13.4)		100 (12.2)		154 (13.8)		130 (16.1)				
	**I was able to reach a member of the team right away for any questions or concerns (n=3039, 95.81%)**														.32^a^
		Total	59 (100)		261 (100)		813 (100)		1106 (100)		800 (100)				
		Agree	54 (91.5)		210 (80.5)		664 (81.7)		899 (81.3)		643 (80.4)				
		Disagree	5 (8.5)		51 (19.5)		149 (18.3)		207 (18.7)		157 (19.6)				
	**The team promptly responded to my needs (n=3038, 95.78%)**														.26^a^
		Total	59 (100)		259 (100)		815 (100)		1105 (100)		800 (100)				
		Agree	55 (93.2)		224 (86.5)		712 (87.4)		943 (85.3)		678 (84.8)				
		Disagree	4 (6.8)		35 (13.5)		103 (12.6)		162 (14.7)		122 (15.3)				
	**The team explained when I should seek medical attention (n=3032, 95.59%)**														<.001^a^
		Total	59 (100)		259 (100)		815 (100)		1105 (100)		794 (100)				
		Agree	53 (89.8)		229 (88.4)		704 (86.4)		921 (83.4)		627 (79)				
		Disagree	6 (10.2)		30 (11.6)		111 (13.6)		184 (16.6)		167 (21)				
	**The educational materials provided by the team were useful (ie, information packets, booklets, pamphlets, etc.), (n=3054, 96.28%)**														.49^a^
		Total	59 (100)		261 (100)		822 (100)		1112 (100)		800 (100)				
		Agree	50 (84.7)		224 (85.8)		679 (82.6)		947 (85.2)		683 (85.4)				
		Disagree	9 (15.3)		37 (14.2)		143 (17.4)		165 (14.8)		117 (14.6)				
**Overall experience**															
	**I felt ready to leave the Remote Patient Monitoring program (n=3077, 97.01%)**														.17^a^
		Total	59 (100)		260 (100)		823 (100)		1116 (100)		820 (100)				
		Agree	56 (94.9)		243 (93.5)		761 (92.5)		1057 (94.7)		778 (94.9)				
		Disagree	2 (3.4)		17 (6.5)		62 (7.5)		59 (5.3)		42 (5.1)				
	**The team treated me with courtesy and respect (n=3070, 96.78%)**														.11^a^
		Total	59 (100)		259 (100)		817 (100)		1119 (100)		816 (100)				
		Agree	57 (96.6)		251 (96.9)		783 (95.8)		1092 (97.6)		799 (97.9)				
		Disagree	2 (3.4)		8 (3.1)		34 (4.2)		27 (2.4)		17 (2.1)				
	**The staff worked well together to care for me (n=3063, 96.56%)**														.57^a^
		Total	58 (100)		260 (100)		819 (100)		1114 (100)		812 (100)				
		Agree	52 (89.7)		238 (91.5)		759 (92.7)		1024 (91.9)		735 (90.5)				
		Disagree	6 (10.3)		22 (8.5)		60 (7.3)		90 (8.1)		77 (9.5)				
	**I would recommend the Remote Patient Monitoring Program to others with a similar health condition(s), (n=3068, 96.72%)**														.62^a^
		Total	59 (100)		259 (100)		818 (100)		1117 (100)		815 (100)				
		Agree	52 (88.1)		240 (92.7)		757 (92.5)		1043 (93.4)		754 (92.5)				
		Disagree	7 (11.9)		19 (7.3)		61 (7.5)		74 (6.6)		61 (7.5)				
	**Overall, I am satisfied with the Remote Patient Monitoring Program (n=3070, 96.78%)**														.98^a^
		Total	59 (100)		261 (100)		817 (100)		1118 (100)		815				
		Agree	55 (93.2)		245 (93.9)		766 (93.8)		1048 (93.7)		759 (93.1)				
		Disagree	4 (6.8)		16 (6.1)		51 (6.2)		70 (6.3)		56 (6.9)				

^a^Chi-square *P* value.

**Table 6:** Remote patient monitoring program patient experience survey questions and responses by patient marital status.

Questions (number of respondents)			Married (n=2401), n (%)	Single (n=262), n (%)	Previously married (n=506), n (%)	*P* value	
**Comfort**							
	**The Remote Patient Monitoring Program helped me feel comfortable managing my health at home (n=3125, 98.61%)**						.54^a^
		Total	2369 (100)	258 (100)	498		
		Agree	2113 (89.2)	231 (89.5)	436 (87.6)		
		Disagree	256 (10.8)	27 (10.5)	62 (12.4)		
**Equipment**							
	**The team explained how to use the equipment (n=3096, 97.7%)**						.23^a^
		Total	2349 (100)	258 (100)	489 (100)		
		Agree	2086 (88.8)	220 (85.3)	435 (89)		
		Disagree	263 (11.2)	38 (14.7)	54 (11)		
	**The medical equipment was easy to use (n=3076, 97.07%)**						.20^a^
		Total	2336 (100)	254 (100)	486 (100)		
		Agree	2179 (93.23)	233 (91.7)	443 (91.2)		
		Disagree	157 (6.7)	21 (8.3)	43 (8.8)		
	**The equipment helped in my care at home (n=3073, 96.97%)**						.03^a^
		Total	2330 (100)	259 (100)	487 (100)		
		Agree	2038 (87.5)	209 (81.6)	427 (87.7)		
		Disagree	292 (12.5)	47 (18.4)	60 (12.3)		
	**I felt comfortable interacting with the team by phone or tablet (n=3078, 97.13%)**						.57^a^
		Total	2332 (100)	257 (100)	489 (100)		
		Agree	2194 (94.1)	238 (92.6)	462 (94.5)		
		Disagree	138 (5.9)	19 (7.4)	27 (5.5)		
**Communication**							
	**The team explained things to me in a way that was easy to understand (n=3076, 97.07%)**						.75^a^
		Total	2336 (100)	256 (100)	484 (100)		
		Agree	2175 (93.1)	238 (93)	446 (92.1)		
		Disagree	161 (6.9)	18 (7)	38 (7.9)		
	**The team listened to my concerns (n=3062, 96.62%)**						.047^a^
		Total	2328 (100)	251 (100)	483 (100)		
		Agree	2097 (90.1)	219 (87.3)	448 (92.8)		
		Disagree	231 (9.9)	32 (12.7)	35 (7.2)		
	**The team kept me informed about my care plan (n=3060, 96.56%)**						.50^a^
		Total	2323 (100)	254 (100)	482 (100)		
		Agree	2002 (86.2)	213 (83.9)	420 (87)		
		Disagree	321 (13.8)	41 (16.1)	63 (13)		
	**I was able to reach a member of the team right away for any questions or concerns (n=3036, 95.8%)**						.61^a^
		Total	2303 (100)	253 (100)	480 (100)		
		Agree	1863 (80.9)	209 (82.6)	396 (82.5)		
		Disagree	440 (19.1)	44 (17.4)	84 (17.5)		
	**The team promptly responded to my needs (n=3035, 95.77%)**						.67^a^
		Total	2304 (100)	251 (100)	480 (100)		
		Agree	1975 (85.7)	220 (87.6)	415 (86.5)		
		Disagree	329 (14.3)	31 (12.4)	65 (13.5)		
	**The team explained when I should seek medical attention (n=3029, 95.58%)**						.96^a^
		Total	2304 (100)	250 (100)	475 (100)		
		Agree	1925 (83.6)	208 (83.2)	399 (84)		
		Disagree	379 (16.4)	42 (16.8)	76 (16)		
	**The educational materials provided by the team were useful (ie, information packets, booklets, pamphlets, etc.), (n=3051, 96.28%)**						.24^a^
		Total	2316 (100)	255 (100)	480 (100)		
		Agree	1971 (85.1)	207 (81.2)	403 (84)		
		Disagree	345 (14.9)	48 (18.8)	77 (16)		
**Overall experience**							
	**I felt ready to leave the Remote Patient Monitoring program (n=3074, 97%)**						.06^a^
		Total	2333 (100)	254 (100)	487 (100)		
		Agree	2208 (94.6)	236 (92.9)	448 (92)		
		Disagree	125 (5.4)	18 (7.1)	39 (8)		
	**The team treated me with courtesy and respect (n=3067, 96.78%)**						.74^a^
		Total	2327 (100)	254 (100)	486 (100)		
		Agree	2263 (97.2)	245 (96.5)	471 (96.9)		
		Disagree	64 (2.8)	9 (3.5)	15 (3.1)		
	**The staff worked well together to care for me (n=3060, 96.56%)**						.09^a^
		Total	2325 (100)	252 (100)	483 (100)		
		Agree	2141 (92.1)	222 (88.1)	442 (91.5)		
		Disagree	184 (7.9)	30 (11.9)	41 (8.5)		
	**I would recommend the Remote Patient Monitoring Program to others with a similar health condition(s), (n=3065, 96.72%)**						.22^a^
		Total	2327 (100)	252 (100)	486 (100)		
		Agree	2166 (93.1)	227 (90.1)	450 (92.6)		
		Disagree	161 (6.9)	25 (9.9)	36 (7.4)		
	**Overall, I am satisfied with the Remote Patient Monitoring Program (n=3067, 96.78%)**						.03^a^
		Total	2329 (100)	252 (100)	486 (100)		
		Agree	2188 (93.9)	226 (89.7)	456 (93.8)		
		Disagree	141 (6.1)	26 (10.3)	30 (6.2)		

^a^Chi-square *P* value.

**Table 7:** Remote patient monitoring program patient experience survey questions and responses by patient education level.

Questions (number of respondents)			High School or less (n=580), n (%)	Associate or some college (n=1055), n (%)	Bachelor’s degree (n=715), n (%)	Master’s or more (n=494), n (%)	*P* value	
**Comfort**								
	**The Remote Patient Monitoring Program helped me feel comfortable managing my health at home (n=2806, 98.66%)**							.64^a^
		Total	571 (100)	1040 (100)	705 (100)	490 (100)		
		Agree	510 (89.3)	916 (88.1)	621 (88.1)	441 (90)		
		Disagree	61 (10.7)	124 (11.9)	84 (11.9)	49 (10)		
**Equipment**								
	**The team explained how to use the equipment (n=2784, 97.89%)**							.26^a^
		Total	568 (100)	1032 (100)	696 (100)	488 (100)		
		Agree	509 (89.6)	929 (90)	620 (89.1)	423 (86.7)		
		Disagree	59 (10.4)	103 (9)	76 (10.9)	65 (13.3)		
	**The medical equipment was easy to use (n=2765, 97.22%)**							.32^a^
		Total	561 (100)	1023 (100)	695 (100)	482 (100)		
		Agree	515 (91.8)	956 (93.4)	649 (93.5)	445 (91.4)		
		Disagree	46 (8.2)	67 (6.6)	45 (6.5)	42 (8.6)		
	**The equipment helped in my care at home (n=2763, 97.15%)**							.02^a^
		Total	563 (100)	1023 (100)	695 (100)	482 (100)		
		Agree	500 (88.8)	884 (86.4)	582 (83.7)	430 (89.2)		
		Disagree	63 (11.2)	139 (13.6)	113 (16.3)	52 (10.8)		
	**I felt comfortable interacting with the team by phone or tablet (n=2767, 97.29%)**							.97^a^
		Total	563 (100)	1023 (100)	698 (100)	483 (100)		
		Agree	528 (93.8)	964 (94.2)	658 (94.3)	456 (94.4)		
		Disagree	35 (6.2)	59 (5.8)	40 (5.7)	27 (5.6)		
**Communication**								
	**The team explained things to me in a way that was easy to understand (n=2754, 96.84%)**							.65^a^
		Total	562 (100)	1030 (100)	694 (100)	481 (100)		
		Agree	522 (92.9)	966 (93.8)	644 (92.8)	443 (92.1)		
		Disagree	40 (7.1)	64 (6.2)	50 (7.2)	38 (7.9)		
	**The team listened to my concerns (n=2754, 96.84%)**							.21^a^
		Total	559 (100)	1027 (100)	689 (100)	479 (100)		
		Agree	515 (92.1)	930 (90.6)	611 (88.7)	429 (89.6)		
		Disagree	44 (7.9)	97 (9.4)	78 (11.3)	50 (10.4)		
	**The team kept me informed about my care plan (n=2756, 96.91%)**							.02^a^
		Total	561 (100)	1021 (100)	694 (100)	480 (100)		
		Agree	502 (89.5)	882 (86.4)	580 (83.6)	404 (84.2)		
		Disagree	59 (10.5)	139 (13.6)	114 (16.4)	76 (15.8)		
	**I was able to reach a member of the team right away for any questions or concerns (n=2730, 95.99%)**							.23^a^
		Total	555 (100)	1022 (100)	678 (100)	475 (100)		
		Agree	465 (83.8)	822 (80.4)	539 (79.5)	390 (82.1)		
		Disagree	90 (16.2)	200 (19.6)	139 (20.5)	85 (17.9)		
	**The team promptly responded to my needs (n=2729, 95.96%)**							.75^a^
		Total	555 (100)	1015 (100)	684 (100)	475 (100)		
		Agree	483 (87)	865 (85.2)	588 (86)	412 (86.7)		
		Disagree	72 (13)	150 (14.8)	96 (14)	63 (13.3)		
	**The team explained when I should seek medical attention (n=2724, 95.78%)**							.32^a^
		Total	552 (100)	1016 (100)	681 (100)	475 (100)		
		Agree	467 (84.6)	854 (84.1)	567 (83.3)	383 (80.6)		
		Disagree	85 (15.4)	162 (15.9)	114 (16.7)	92 (19.4)		
	**The educational materials provided by the team were useful (ie, information packets, booklets, pamphlets, etc.), (n=2746, 96.55%)**							.01^a^
		Total	558 (100)	1025 (100)	687 (100)	476 (100)		
		Agree	479 (85.8)	891 (86.9)	561 (81.7)	394 (82.8)		
		Disagree	79 (14.2)	134 (13.1)	126 (18.3)	82 (17.2)		
**Overall experience**								
	**I felt ready to leave the Remote Patient Monitoring program (n=2766, 97.26%)**							.29^a^
		Total	561 (100)	1028 (100)	697 (100)	480 (100)		
		Agree	528 (94.1)	971 (94.5)	661 (94.8)	443 (92.3)		
		Disagree	33 (5.9)	57 (5.5)	36 (5.2)	37 (7.7)		
	**The team treated me with courtesy and respect (n=2762, 97.12%)**							.78^a^
		Total	557 (100)	1029 (100)	697 (100)	479 (100)		
		Agree	539 (96.8)	1001 (97.3)	681 (97.7)	467 (97.5)		
		Disagree	18 (3.2)	28 (2.7)	16 (2.3)	12 (2.5)		
	**The staff worked well together to care for me (n=2753, 96.8%)**							.04^a^
		Total	555 (100)	1025 (100)	692 (100)	481 (100)		
		Agree	524 (94.4)	934 (91.12)	623 (90)	438 (91.1)		
		Disagree	31 (5.6)	91 (8.88)	69 (10)	43 (8.9)		
	**I would recommend the Remote Patient Monitoring Program to others with a similar health condition(s), (n=2758, 96.98%)**							.38^a^
		Total	561 (100)	1024 (100)	692 (100)	481 (100)		
		Agree	529 (94.3)	946 (92.4)	636 (91.9)	443 (92.1)		
		Disagree	32 (5.7)	78 (7.6)	56 (8.1)	38 (7.9)		
	**Overall, I am satisfied with the Remote Patient Monitoring Program (n=2760, 97.05%)**							.69^a^
		Total	556 (100)	1026 (100)	697 (100)	481 (100)		
		Agree	523 (94.1)	960 (93.6)	653 (93.7)	444 (92.3)		
		Disagree	33 (5.9)	66 (6.4)	44 (6.3)	37 (7.7)		

^a^Chi-square *P* value.

### Patient Comments

Written responses to the 2 open-ended questions of the survey allowed the patient to comment about aspects of the RPM program that either most impressed or disappointed them. The random selection of comments is presented in Textbox 2. Of the 3172 participants, 74.24% (2355/3172) provided positive (“impressed”) comments and 61.54% (1952/3172) provided negative (“disappointed”) comments. Notably, of the comments related to what disappointed them, 31.35% (612/1952) of respondents wrote comments like “I cannot think of any that disappointed me,” “all was well,” “absolutely nothing,” and “I wasn’t disappointed.” These can be considered positive comments; thus, only 42.24% (1340/3172) were reported as negative comments.

**Random selection of patient comments about their Remote Patient Monitoring program experience.**

**What impressed you?**

- “I was most impress with the level of communication and the understanding of my condition.”
- “The technology of the system. Was able to stay home and people kept track of my vitals and health.”
- “Excellent communications by nurses. They were friendly and caring.”
- “The entire process was seamless: technology worked as described, delivery and return went well, medical advice and suppress was proactive and very helpful. I would strongly recommend the remote monitoring program to anyone.”
- “How fast the team responded to my Covid infection.”
- “Once, when my blood pressure and pulse reading was submitted via the included cell phone, I almost immediately received a chat message on the phone asking me to re-do the reading and submit the new reading. I was impressed that my vital sign readings were being monitored in real time.”
- “The equipment was easy to use, and I felt like if I was not sure that the team would answer my questions.”

**What disappointed you?**

- “I didn’t care for the early morning checks even though they were very necessary. The problem with early checks was only a struggle because of my condition, fatigue, and lack of sleep.”
- “Nothing except hard to balance on scale.”
- “Delivery of equipment and startup could have been a day or two sooner. Realize that weekend and rural location impacted this.”
- “I was not disappointed in any way.”
- “There was a delay of several days between the time I agreed to participate and when the equipment actually arrived. I was monitored because I had COVID, and by the time the equipment arrived, I had received monoclonal antibodies and was very much on the mend. There being a weekend seemed to slow down the delivery of the equipment.”
- “The morning window could have been a little later or longer. I am not a morning person, so I had to get up. It was not a problem, but I would have liked to sleep later.”
- “Difficult getting Wi-Fi to work.”

## Discussion

### Principal Findings

This survey-based analysis established that there is a high level of patient satisfaction with the Mayo Clinic’s RPM program. A major strength of this study is the large sample size of 3128 patients, and the RPM program’s reach and representation across 3 major US geographic regions and within tertiary and community-based clinical practices. Of the analytic cohort, 88.97% (2783/3128) agreed or strongly agreed that the program helped them feel comfortable managing their health at home. Furthermore, 94.09% (2895/3077) stated that they were satisfied with the program and felt ready to graduate upon reaching their goals. In addition, the confidence of patients in this model of care was confirmed by 92.76% (2846/3068) of the participants who would recommend the program to people with similar conditions.

High-quality health care can be achieved through delivery using an RPM model for a wide range of clinical conditions [14,23-28]. Reductions in health care costs, hospital readmission, and increased access to care with better clinical outcomes and patient satisfaction are some of the potential benefits associated with this innovative strategy of offering health care outside of traditional clinical settings [16,19,21,29-31]. Furthermore, patients expressed feeling more informed about their condition and more connected with their providers when continuous monitoring was provided [32,33]. Similarly, health care providers have experienced a high level of acceptance in the inclusion of RPM as part of their daily practice [19,34]. Thus, especially among patients with chronic conditions, RPM allows providers to better understand how their patients manage their condition between clinic visits, promptly recognize any early signs of adverse health trends and implement needed diagnostics, treatment, or lifestyle changes to optimize care [3,35,36].

Besides the quality of the technology and medical devices, their ease of use influences the patient’s experience as well as their clinical outcomes [37]. Overall, the patients had favorable experiences with the technology and devices used in the RPM program. Furthermore, over 88.54% (2744/3099) of participants agreed or strongly agreed that the staff explained how to use the devices, the technology was easy to use, and felt comfortable interacting with the team through this technology. Interestingly, the youngest age group was much less in agreement that the equipment helped them manage their health from home. This may reflect a lower disease acuity and fewer comorbidities associated with overall health. These findings call into question the value of using the full technology package for the RPM program in this cohort.

Technology use in the older adult population has been a substantial limitation in adopting telehealth and web-based care [38,39]. Identifying the factors that influence the rate of technology acceptance and digital literacy in this population helps to overcome this limitation. Thus, understanding the perspectives and concerns of older patients and considering them when designing medical devices may increase the likelihood of acceptance of the technology [40]. Although the overall population in this cohort had a median age of 66.5 years, the high patient acceptance of our RPM program differs from what was previously reported in the literature [39]. We attribute iterative improvements in the technology user experience, system setup, and the nurse welcome call, and education has been demonstrated to overcome the main concerns reported by this population, such as the level of comfort using the devices, the ability to install the equipment, and some difficulties completing the assigned task faster than younger patients [29,41].

Self-care is vital for maintaining a good quality of life, particularly in patients with chronic conditions [5,42]. Hence, RPM staff must be able to immerse their patients in self-management strategies. Thus, communication between patients, nurses, and providers is a critical aspect of this type of care delivery model [43]. A high percentage of the patients agreed or strongly agreed that the health care team operating the RPM program had excellent communication skills, including the ability to explain things to them in a comprehensible way, keep them informed about the health care plan, and listen to their concerns. However, the percentage of patients with a neutral position was notable for the speed of the team in answering any questions or concerns, explaining when patients should seek medical attention, and the utility of the educational material provided by the team. Although only 15.7% (477/3039) or fewer of patients maintained this neutral posture, these are essential factors that should be addressed in future RPM quality improvement projects.

In contrast, in acute conditions, patients’ engagement with RPM can be reduced by physical health factors such as the severity of the symptoms that limit equipment management, team interaction, and completion of the task assigned [17]. These situations must be identified before patients’ enrollment in the program to determine the need for additional support. Therefore, the program’s success will depend on offering individualized services to cover patients’ specific needs that lead them to feel reassured and supported. In addition, some studies have reported that patients’ self-monitoring and staying in contact with providers increases their confidence and improves their accountability, engagement, and participation in their health care [44,45].

Furthermore, the RPM program represents a good resource for participant education and an excellent way to encourage participants to establish targets and achieve their health goals. It was notable that patients with the lowest education level reported the highest level of agreement that “the team kept me informed about my care plan” and with “the educational materials provided by the team were useful.” To our knowledge, this has not been previously reported, and it highlights the potential for RPM programs to address this critical social determinant of health, as well as the need for more dedicated research to assess the value of RPM in this cohort.

Although many advantages have been associated with using RPM care models [8,14,20,26,38], some disadvantages have been reported by both patients and providers. Thus, patients’ privacy, financial burden, data inaccuracy, and increasing patients’ anxiety are potential adverse events reported for RPM [45,46]. Some of the most frequent concerns were related to equipment delivery and return processes at the start and end of the program. In addition, patients reported problems with blood pressure cuff size, measurement inaccuracy, especially with the thermometer, and issues with their connection. The identification of these undesirable situations can direct efforts toward improvement.

Overall, the patient’s level of satisfaction and engagement plays a crucial role in the effectiveness of the service and application of optimization strategies.

There were limitations to the RPM program analysis. Although a large number of patients responded to the survey, the low response rate limits the extrapolation of these results to the entire RPM population. This may be explained by the nonresponse risk of bias associated with survey studies [47]. Furthermore, using email to deliver the survey will miss those patients without an account, and it could favor results toward those more technically adept respondents who have a positive impression of technology-based care approaches such as RPM. In addition, the analytic cohort was predominantly comprised of non-Hispanic, White patients, and further research is needed to understand whether there are differences in RPM program use and satisfaction within underrepresented minority group populations. Finally, the subjectivity in the interpretation of the open-ended questions and survey results is a potential cause of bias associated with descriptive, survey-based analyses.

### Conclusions

The effectiveness of RPM in delivering high-quality health care has been established across a broad range of acute and chronic clinical conditions. This analysis demonstrated a high level of satisfaction experienced by patients enrolled in our multisite, multiregional RPM program. The service uses digital health technologies to facilitate monitoring of patient vital signs and symptoms by a clinical care team. However, further enhancements should be considered to address the concerns reported by a low percentage of patients concerning device delivery, measurement accuracy, and some aspects of the health education information provided to the user.

## Abbreviations

RPM: remote patient monitoring

## Data Availability

The inclusion of protected health information can be made available after deidentification upon reasonable request to the corresponding author (MJM).
